# Condition Assessment of Natural Ester–Mineral Oil Mixture Due to Transformer Retrofilling via Sensing Dielectric Properties

**DOI:** 10.3390/s23146440

**Published:** 2023-07-16

**Authors:** Hesham S. Karaman, Diaa-Eldin A. Mansour, Matti Lehtonen, Mohamed M. F. Darwish

**Affiliations:** 1 Department of Electrical Engineering, Faculty of Engineering at Shoubra, Benha University, Cairo 11629, Egypt; hesham.said@feng.bu.edu.eg; 2 Department of Electrical Power Engineering, Egypt-Japan University of Science and Technology (E-JUST), Alexandria 21934, Egypt; diaa.mansour@ejust.edu.eg; 3 Department of Electrical Power and Machines Engineering, Faculty of Engineering, Tanta University, Tanta 31511, Egypt; 4 Department of Electrical Engineering and Automation, School of Electrical Engineering, Aalto University, 02150 Espoo, Finland

**Keywords:** mineral oil, natural ester oil, power transformer, dielectric properties measurement, dynamic viscosity, condition assessment, mixture oil, accelerated aging, diagnostic process

## Abstract

Mineral oil (MO) is the most popular insulating liquid that is used as an insulating and cooling medium in electrical power transformers. Indeed, for green energy and environmental protection requirements, many researchers introduced other oil types to study the various characteristics of alternative insulating oils using advanced diagnostic tools. In this regard, natural ester oil (NEO) can be considered an attractive substitute for MO. Although NEO has a high viscosity and high dielectric loss, it presents fire safety and environmental advantages over mineral oil. Therefore, the retrofilling of aged MO with fresh NEO is highly recommended for power transformers from an environmental viewpoint. In this study, two accelerated aging processes were applied to MO for 6 and 12 days to simulate MO in service for 6 and 12 years. Moreover, these aged oils were mixed with 80% and 90% fresh NEO. The dielectric strength, relative permittivity, and dissipation factor were sensed using a LCR meter and oil tester devices for all prepared samples to support the condition assessment performance of the oil mixtures. In addition, the electric field distribution was analyzed for a power transformer using the oil mixtures. Furthermore, the dynamic viscosity was measured for all insulating oil samples at different temperatures. From the obtained results, the sample obtained by mixing 90% natural ester oil with 10% mineral oil aged for 6 days is considered superior and achieves an improvement in dielectric strength and relative permittivity by approximately 43% and 48%, respectively, compared to fresh mineral oil. However, the dissipation factor was increased by approximately 20% but was at an acceptable limit. On the other hand, for the same oil sample, due to the higher molecular weight of the NEO, the viscosities of all mixtures were at a higher level than the mineral oil.

## 1. Introduction

Power transformers have an intrinsic role in the electrical power network. Not only at the generation stations as step-up substations but also at the consumers as step-down substations. These power transformers may be dry or oil-type transformers. In the oil-type power transformers, oil is used as an insulating and cooling medium [1,2]. The popular insulating oil used in oil-filled transformers is mineral oil (MO). Mineral oil has significant toxic effects when spilling into the soil and waterways that include the following. (1) MO has low biodegradability with harmful environmental effects. This indicates that just a small portion of oil self-degrades after being released into the environment [3]. One kilogram of oil leakage waste from a transformer renders 5 million liters of water unfit for consumption [4]. (2) MO has low flash and fire points. Once a fire hazard occurs, polycyclic aromatic hydrocarbons are released as combustion byproducts, which are toxic byproducts with an extreme threat to the environment [5]. Further, burning MO releases toxic gases into the environment [4] and produces heavy and dark smoke. (3) The cost of cleaning up oil spills is often quite high; big environmental utilities spend millions of dollars each year to do so in order to minimize their consequences. (4) MO has inadequate moisture tolerance and poor performance at high temperatures. (5) MO is a non-renewable fossil resource that might run out in the next several decades [6].

Due to the above-mentioned limitations of MO, great attention must be paid to replacing MO with an alternative oil that permits a higher degree of sustainability, is environmentally friendly, and has the same insulating and cooling properties [7]. In the last two decades, there has been an ascension in the usage of natural ester oil (NEO) as a strong alternative to mineral oil because of its high biodegradability [8], which means it easily decomposes into the soil. Hence, many researchers are oriented to study the different properties, characterizations, health index, and condition assessment of natural ester oil using various optical spectroscopy techniques [9,10,11,12]. Moreover, the NEO properties have been compared to that of MO [13,14]. On the other hand, there are some studies interested in the properties of mineral oil mixed with another insulating liquid. Perrier et al. concluded that a mixture of mineral oil with approximately 20% synthetic ester oil achieved an improvement in the dielectric properties and the aging stability without viscosity degradation compared to mineral oil alone [15]. Nadolny et al. studied the thermal properties of different mixture ratios of mineral and ester oils. According to this study, the optimal ratio of ester oil is 5% at which the heat transferability is highest and thus provides the best cooling performance for the transformer [16]. Suwarno et al. concluded that an increase in the percentage of ester contents in mineral oil provides an increase in the mixture’s breakdown voltage, although the dissipation factor is slightly degraded [17]. In [18], 15% and 20% ester oil ratios provided a better dielectric behavior than other samples in this study. Toudja et al. reported that the measured charging current, resistivity, and mobility of different samples show that a mixture of 85% mineral oil with 15% plant oil is the most excellent mixture [19]. Dombek et al. presented that, with an increase in the ester contents in synthetic ester/mineral oil mixtures, the flash point and fire point determined with the open cup technique increased [20]. In [21], Beroual et al. reported that, under AC or DC applied voltage, Jatropha methyl ester oil has a higher breakdown voltage than MO, and the addition of this oil to MO extensively upgrades the breakdown voltage of the obtained mixture. Further, Ref. [22] proposes a prediction model of the transformer oil breakdown voltage in the existence of diverse barrier effects for point/plane gap systems with an AC supply voltage using a Box–Behnken design. Recently, Dixit et al. investigated the temperature distribution within the whole structure winding as well as the natural cooling category distribution transformer underneath a retrofilling with natural ester [23]. In addition, a comprehensive literature review on the mixture of MO with other alternative dielectric fluids such as natural or synthetic esters is presented [1]. This is helpful for utilities, researchers, as well as transformer owners that are interested in ester liquids besides retrofilling aspects.

As revealed in the above-mentioned literature, most of the existing studies have poor condition assessment and diagnostic tools of the dielectric properties for power transformer oil mixtures under aging processes. To solve this issue, this research work seeks to fill this literature gap by improving the condition assessment and diagnostic performance of the power transformer, specifically the properties of the oil inside that should be enhanced to produce a good insulating and cooling medium under aging. Therefore, replacing or retrofilling aged MO with fresh NEO is one of the approaches to improve power transformer performance. Due to the presence of some mineral oils absorbed by the pressboard between the transformer windings and at the bottom walls of the tank, the oil inside the tank after the replacement process is a mixture of fresh NEO with some quantities of aged mineral oil. The remaining quantities of MO range between 7% and 20% [1,24,25]. In [24], the breakdown voltage was evaluated for vegetable oil and synthetic oil mixed with MO. When using 20% MO, the drop in the AC breakdown voltage was approximately 3% and 7% for vegetable oil and synthetic oil, respectively. In [25], the AC breakdown strength of vegetable oils mixed with 10% and 30% MO was investigated. It was found that there is a slight decrement in AC breakdown strength after mixing with mineral oil compared to pure vegetable oil. This decrement attained approximately 6% and 13% for coconut oil and soybean oil, respectively, when mixed with 30% MO. Previous studies on transformer retrofilling considered only the breakdown voltage for investigation. In addition, these studies used MO in the fresh state, which does not represent the actual MO state during the retrofilling process, where MO is usually aged when retrofilling.

Therefore, in this paper, the mixture of NEO with aged MO was investigated to represent the actual condition when retrofilling. Two percentages of aged MO were considered, 10% and 20%, at different aging periods. Two different aging periods were considered, 6 days and 12 days, simulated with an accelerated aging process to be equivalent to 6 years and 12 years in the actual field service. The dielectric properties, breakdown strength, relative permittivity, and dielectric losses were sensed using a LCR meter and oil tester devices for all prepared samples to evaluate the mixtures’ dielectric properties. Also, Weibull distribution analysis was introduced to evaluate the probability of breakdown for the different mixtures. The viscosity of all prepared samples was measured to investigate the dynamic performance of the different mixtures. In addition, the physical mechanism is discussed to present the dielectric and dynamic performance of ester oil mixed with mineral oil in a power transformer. Finally, an electrostatic model of an oil-filled distribution transformer is developed with COMSOL Multiphysics Software to illustrate the electric field distribution inside the transformer in the case of refilling the transformer with natural ester oil (for the best retrofilling oil sample).

## 2. Oil Specifications and Experimental Proceedings

In this section, the specifications of NEO and MO are presented. Then, the MO aging process and the preparation of samples are described. Finally, the sensing system used for evaluating the dielectric properties in the laboratory is detailed.

### 2.1. Oil Specifications

The obtained samples in this study were prepared by mixing fresh natural ester oil and mineral oil with different percentages, noting that the NEO and MO are commercial types and treated by the manufacturer. Moreover, all samples were treated with a vacuum oven in the laboratory to remove the moisture before starting the experimental process. Table 1 presents the specifications of both oils based on the datasheets provided by the suppliers.

### 2.2. Aging Process

In order to prepare aged mineral oil that simulates the aged oil in the power transformer in service, an accelerated thermal aging process is required [26]. This process was carried out for the fresh mineral oil using a heating oven. At 120 °C, one day in the oven is equivalent to 380 days, i.e., approximately one year in the field [27], where the base operational transformer oil temperature is 60 °C and the aging rate of oil is doubled when the temperature raises by 7 °C, according to the following Equations (1) and (2) [11]:

(1) $$Aging\;accelerating\;factor=\frac{120\;{}^{\circ}C-60\;{}^{\circ}C}{7\;{}^{\circ}C}=8.57$$

(2) $$Time\;factor=2^{8.57}=380$$

In this study, two different aging periods were considered. These periods were 6 days and 12 days that were equivalent to 6 years and 12 years in the field. The obtained aged mineral oil samples aged for 6 days and 12 days were called MO6D and MO12D, respectively.

### 2.3. Sample Preparation

**Figure 1:**
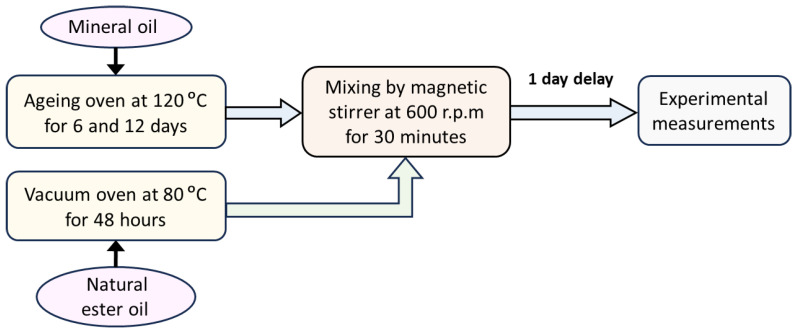
Schematic diagram to illustrate the preparation process of the oil samples.

The mineral oil was aged as mentioned in the previous subsection to simulate the real field. The aged mineral oil was mixed with fresh natural ester oil after heating it in a vacuum oven at 80 °C for 48 h to remove gas bubbles and moisture contents. A magnetic stirrer was used to mix the two types of oils at 600 rpm for 30 min. The prepared oil samples were left for 24 h before the measuring processes as shown in Figure 1.

### 2.4. Dielectric Strength

**Figure 2:**
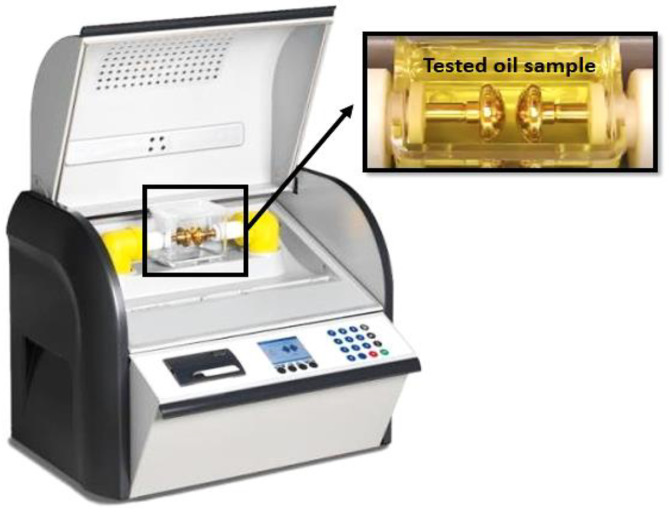
AC breakdown tester for liquid insulations.

The dielectric strength of the transformer oil is the most important characteristic that indicates the quality of its insulation property. Consequently, when the dielectric strength is high, it is an indication of a good quality transformer oil that reflects positively on the transformer’s performance. Therefore, in this study, the prepared oil samples were subjected to a breakdown test using the AC oil tester presented in Figure 2 (from 0 to 100 kV) to evaluate and sense the dielectric strength of each sample. The test proceeded based on the IEC-60156 standard with a 2 kV/s voltage ramp rate and a 2.5 mm separation distance between the mushroom-shape test electrodes. For each sample, ten results were recorded and analyzed with special sensors to perform the Weibull distribution cumulative probability function to provide all probabilities of AC breakdown strength with a small number of tests [28]. The Weibull distribution cumulative probability function for the oil sample *F*(*v*) can be introduced as shown in Equation (3): *v* is the breakdown voltage in kV, *λ* denotes the scale parameter in kV, and *ξ* represents the shape parameter [29].

(3) $$F\left(v\right)=1-e^{-(\frac{v}{\lambda }{)}^{\xi }}$$

### 2.5. Dielectric Properties

Due to the presence of an electric field on the insulating oil, dielectric polarization occurs for its molecules. The ability of polarization under the effect of the applied field can be defined with the expression of the relative permittivity (*ε_r_*) [30]. In the composite insulation system of the oil-filled transformer (oil/paper), the greater the relative permittivity of the insulating oil, the more uniform the electric field at the interface between the oil and the insulating paper. Therefore, increasing the relative permittivity of the insulating oil is preferable for good performance of the transformer [29]. On the other hand, there is another parameter that indicates the insulation system quality. This parameter is the dissipation factor of the insulating liquid (tan *δ*). A high value of the dissipation factor refers to the presence of more existing contaminations and imperfections in the insulating oil [31,32]. Therefore, mitigation of the dissipation factor value provides a positive impact on the insulation system. The relative permittivity and dissipation factor can be estimated from the following Equations (4)–(7); Table 2 defines the different variables in the given equations.

(4) $$\varepsilon_{r}=\varepsilon^{\prime }-j\varepsilon^{\prime \prime }$$

(5) $$\tan\delta =\frac{\varepsilon^{\prime \prime }}{\varepsilon^{\prime }}$$

(6) $$\varepsilon^{\prime }=\frac{dC_{p}}{\varepsilon_{o}A}$$

(7) $$\varepsilon^{\prime \prime }=\frac{d\;}{{2\pi f\varepsilon }_{o}AR_{p}}$$

**Figure 3:**
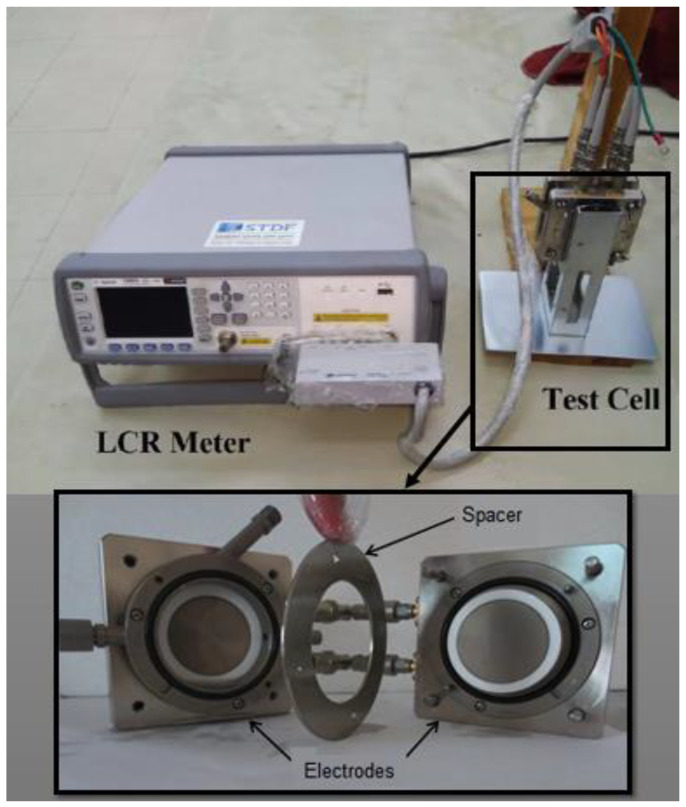
LCR meter and the test cell used for the dielectric measurements.

In order to sense and evaluate the relative permittivity values as well as the dissipation factor of the oil sample, the capacitance (*Cp*) and the resistance (*Rp*) of the oil sample must be measured. The sensing system refers to the experimental measurements in the laboratory used for evaluating the dielectric properties. In this study, the Agilent E4980A Precision LCR meter was employed to sense and measure the *Cp* and *Rp* values using advanced sensors built into the LCR meter under the adjustment of the equivalent circuit parallel model of the LCR meter. Figure 3 presents the LCR meter and the test cell that were used in this test with 0.3 mm gap spacing and 38 mm electrode diameter under a wide range of frequencies from 20 Hz to 1 MHz. After measuring the values of *Cp* and *Rp* of each sample with the LCR meter, the value of *Cp* was applied in Equation (6) to evaluate the real permittivity *ε*′, and the value of *Rp* was employed in Equation (7) to estimate the imaginary permittivity *ε*″. Thus, the obtained values of relative permittivity and the dissipation factor were computed from Equations (4) and (5), respectively.

### 2.6. Dynamic Viscosity Preparation

It is known that the viscosity of any liquid is the resistance of this liquid to flow. The viscosity of the transformer oil is a very important property in oil-filled transformers for providing a good heat-exchange process. Therefore, a lower viscosity of the oil results in a higher circulation speed of the oil and better efficiency of the cooling system. In this study, the Kinematic Viscosity Bath (KV3000) was used to measure the viscosity of the prepared oil samples. This device is used to sense and measure the flow of the liquid under gravity or vacuum at specified controlled temperatures.

## 3. Results and Discussion

### 3.1. Dielectric Strength

**Figure 4:**
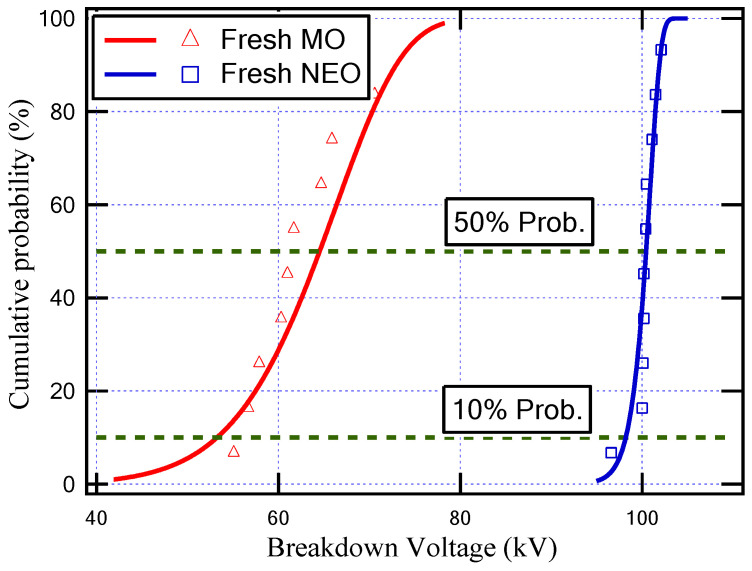
Cumulative probability against breakdown voltage for fresh mineral oil (MO) and fresh natural ester oil (NEO).

The usage of natural ester oil in power transformers not only provides environmental advantages over mineral oil but also provides higher dielectric properties. Figure 4 displays the cumulative probability function for fresh MO and fresh NEO. As shown in Figure 4, the dielectric strength of NEO is higher than that of MO by more than 50%. To study the refilling of the transformer with fresh natural ester oil, six cases of oil testing measurements were introduced in addition to the cases of fresh oils (MO and NEO). These cases are presented in Table 3, where 90% and 80% of NEO mixed with 10% and 20% of aged MO, respectively, at different aging periods are utilized. These percentages are determined based on the expected remaining quantities of MO during the transformer retrofilling process, as specified in previous studies [1,24,25].

**Figure 5:**
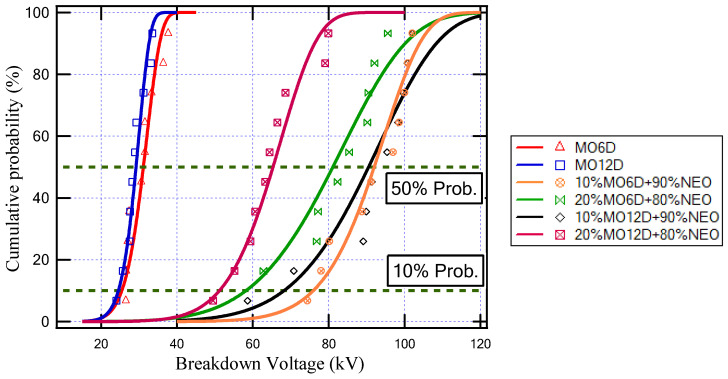
Cumulative probability against breakdown voltage for several aged oil samples.

Figure 5 indicates the cumulative probability function for the oil samples illustrated in Table 3. In addition, Table 4 depicts the shape parameter (ξ), scale parameter (λ), breakdown voltage (BDV) with an AC supply at 50% probability (BDV50%), and BDV at 10% probability (BDV10%) for all insulating oil samples.

The aging of any insulating oil causes a degradation of its dielectric properties due to the thermal stresses affecting the insulating oil bonds. From the obtained results, the aging of mineral oil for 6 days and for 12 days caused a decrease in the breakdown voltage at 50% probability from 64.6 kV to 31.2 kV and 29.2 kV, respectively, while the BDV at 10% probability decreased from 53.3 kV to 25.5 kV and 24.6 kV, respectively. On the other hand, the obtained results show that, due to the increase in the ratio of NEO that fills the transformer, the breakdown strength of the insulating oil inside the transformer increased as presented in Table 4. Furthermore, the oil sample that has a higher BDV is (10% MO6D + 90% NEO) as highlighted in Table 4. This sample achieved an improvement in the BDV by 43% compared to MO and by 195% compared to MO6D, which was attributed to this sample having a larger percentage of NEO and a lower aging period.

### 3.2. Dielectric Properties

**Figure 6:**
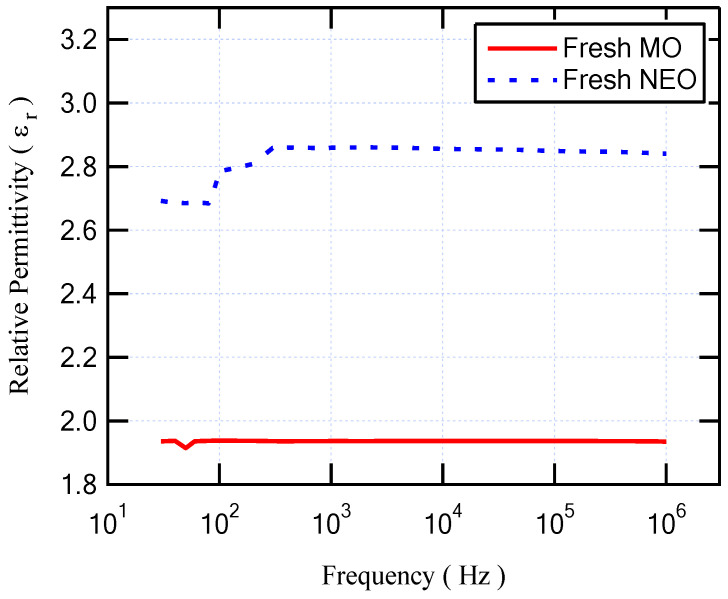
Relative permittivity versus frequency for fresh mineral oil (MO) and fresh natural ester oil (NEO).

**Figure 7:**
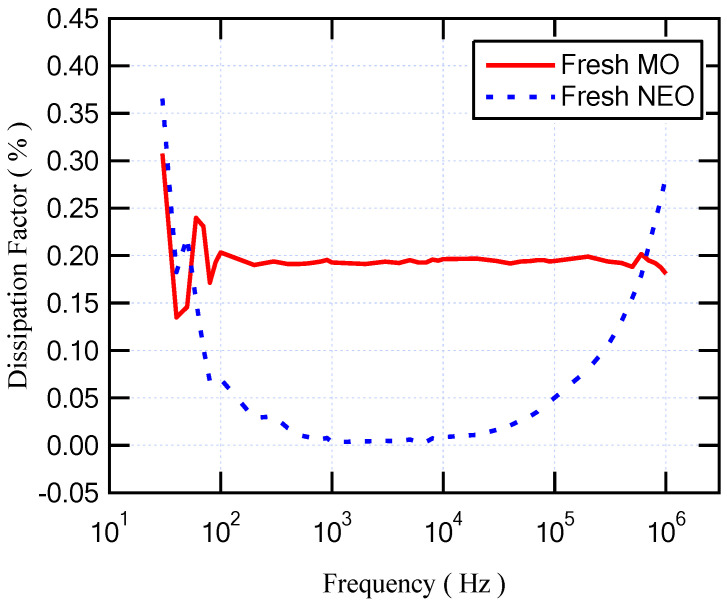
Dissipation factor versus frequency for fresh mineral oil (MO) and fresh natural ester oil (NEO).

**Figure 8:**
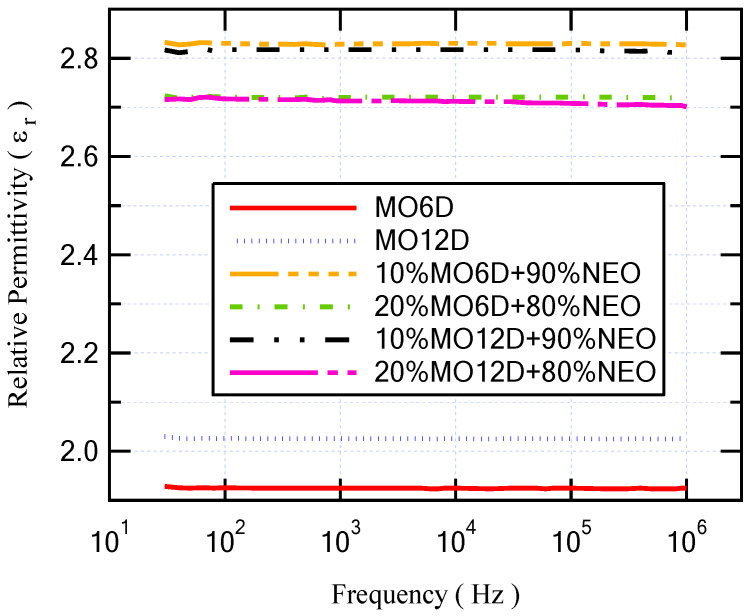
Relative permittivity versus frequency for several aged oil samples.

**Figure 9:**
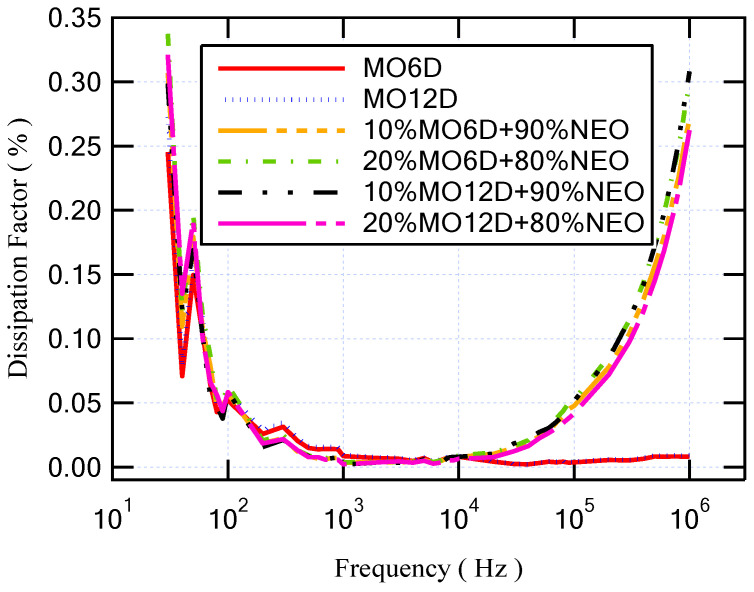
Dissipation factor versus frequency for several aged oil samples.

The Agilent E4980A Precision LCR meter sensed and measured the capacitance and resistance of the different oil samples to evaluate their dielectric parameters at a frequency range from 20 Hz to 1 MHz at room temperature (25 °C). The changing of the dielectric permittivity refers to the ionic and dipolar polarizations that occur via this range of frequencies [33]. Figure 6 shows the relative permittivity versus frequency for fresh MO and fresh NEO. As presented, the relative permittivity of NEO is higher than that of MO for all frequency ranges. Regarding the dissipation factor that is shown in Figure 7, the dissipation factor of MO is lower than that of NEO by approximately 0.074. On the other hand, with regard to the longer period of aging for insulating MO, the relative permittivity was enhanced, but the dissipation factor degraded. This result is similar to that obtained with the mixture of NEO and MO as summarized in Table 5. Moreover, Figure 8 and Figure 9 illustrate the variation in relative permittivity and the dissipation factor with different frequencies, respectively. The oil sample that has a higher BDV is (10% MO6D + 90% NEO) as highlighted in Table 4 and mentioned in the previous subsection. Furthermore, the sample of (10% MO6D + 90% NEO) achieved the best relative permittivity with a value equal to 2.829 compared to all other oil samples. Even though the dissipation factor of this oil sample is not the lowest, it is still under the acceptable value mentioned in IEC 60296.

### 3.3. Dynamic Viscosity Measurement

**Figure 10:**
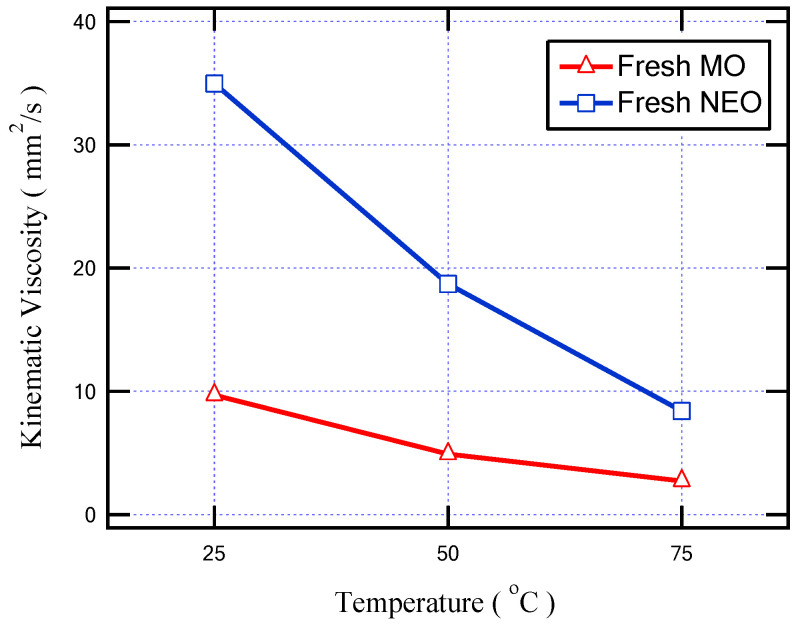
The variation in fresh insulating oil viscosity with temperature increment.

For the enhancement of the cooling property of the insulating oil due to its higher circulation speed, a lower viscosity of the oil is necessary. Figure 10 shows the relationship between viscosity and temperature for fresh NEO and MO. The test was carried out at temperatures of 25, 50, and 75 °C. It is clear that the viscosity of MO is lower than the viscosity of NEO for all tested temperatures. This indicates that the cooling performance of MO is better than that of NEO. This is because the main component of NEO is a triglyceride, and its molecular weight is higher than that of the hydrocarbon chains of MO, so the NEO kinematic viscosity is greater than that of MO [34].

**Figure 11:**
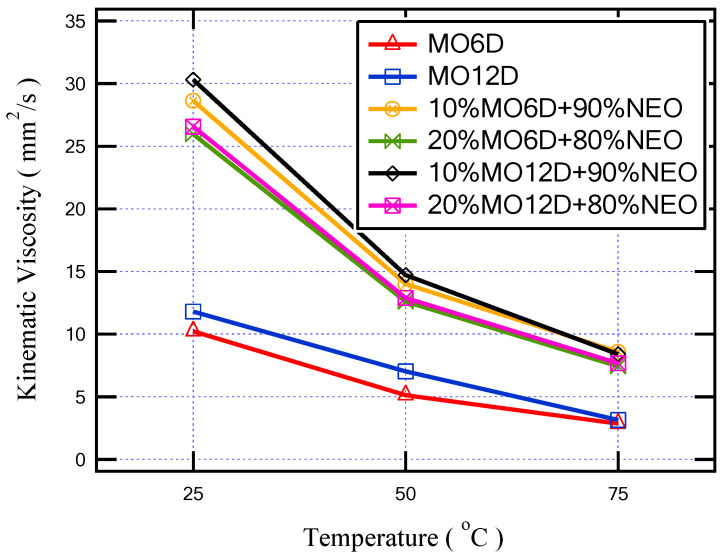
The variation in viscosity with temperature increment for all insulating aged oil samples.

Due to the aging of the mineral oil for 6 and 12 days, as introduced in Figure 11, the oil viscosity increased by approximately 4% and 40%, respectively, compared with fresh MO at 50 °C. On the other hand, due to the transformer retrofilling process with NEO, the oil mixture sample that achieved the lowest viscosity is the sample of (20% MO6D + 80% NEO). This sample provides better cooling performance due to a lower aging period in addition to a lower ratio of NEO. However, the sample of (10% MO6D + 90% NEO), which achieves better dielectric performance as discussed in the previous sections, provides a good viscosity value that is close to the viscosity value of the sample of (20% MO6D + 80% NEO).

## 4. Physical Mechanisms

Regarding the dielectric strength of the insulating oils, the AC-BDV of insulating oil is mainly related to the relative moisture contents. Therefore, the higher saturation moisture content of mixed insulation oil is a major aspect that produces a higher AC-BDV than that of MO at a similar absolute moisture content [35,36]. This concept can be an explanation for the increasing AC-BDV as the percentage of NEO increases in the oil mixture and also with a lower aging period as summarized in Table 4. The higher aging period of MO produces lower dielectric properties for this oil. This can be attributed to the breaking of some MO molecules due to the applied electric field during the transformer’s continuous operation. From the obtained results, for 20% MO, the aging of MO for 12 days achieved an AC-BDV of 65.3 kV, which is lower than 81.1 kV achieved at 6 days, while for 10% MO, the AC-BDV was reduced from 92 kV to 90.2 kV from 6 days to 12 days aging, respectively. Note that the supply of the measured breakdown voltage here is an AC supply; otherwise, from the literature, the NEO performance under impulse waveforms or other fast transients tends to be lower than that of MO, especially if used in barrier-style insulation or homogenous paper oil insulation. Therefore, it can be interesting to study this performance in future work.

On the other hand, the relative permittivity and the dissipation factor of the mixed insulating oil are not only dependent on the percentage of NEO but also on the aging period as presented in Table 5. This dependency relates to the dipole polarization inside the mixed oil [29]. Moreover, the ionic compounds in the aged oil may be dissolved in NEO because of their polar nature; in addition, when the percentage of NEO is increased, this effect becomes prevalent. The increasing dissipation factor of the mixed oil may be due to the aged MO containing a high quantity of polar colloidal components and odorous hydrocarbons [37].

Regarding the dynamic viscosity of the insulating oils, the lower viscosity of MO for all ranges of temperature refers to a better coolant medium. This behavior may be attributed to its lighter hydrocarbon molecular weight than that inside the mixed oil that contains a large amount of NEO’s heavy triglycerides [34].

## 5. Electrostatic Modelling of Oil-Filled Transformer

In this section, an electrostatic model of a 1 MVA, 50 Hz, 22/0.4 kV step-down transformer is simulated using COMSOL Multiphysics Software. The aim of this simulation is to evaluate the distribution of the electrostatic field inside the transformer when using the NEO–MO oil mixtures after the retrofilling process. The case of the oil mixture (10% MO6D + 90% NEO) was considered for the electrostatic field analysis, as it exhibited superior dielectric properties.

COMSOL software is utilized to resolve the desired non-uniform field via the numerical finite element method to effectively model the electrostatic field inside any part of the transformer, specifically the sharp edges and interface points. The input parameters for this model involve the measured r.m.s rates of the AC breakdown voltage followed by the measured dielectric loss and permittivity of the prepared oil samples (NEO–MO mixture). In addition, the other detailed model parameters of the transformer mentioned in Table 6 and the datasheet parameters of numerous oil types are extracted from real experimental data. The computation of the two-dimensional (2D), axis-symmetric field distribution can be exploited via the finite element method (FEM) through the interface area between the winding and NEO–MO mixture. The mechanism of the COMSOL operation depends on the field region that should first be divided into smaller triangle elements to shrink the energy throughout the wide field area of interest by applying the numerical FEM [38]. Once a stationary electric field is utilized for a liquid dielectric substance, the Equations (8) and (9) deliver the electrical energy stored inside the full volume of the area under study, taking into consideration the cylindrical model geometry as well as the Laplacian field Equation (8) for static field [39].

(8) $$\nabla^{2\;}V=0$$

(9) $$\vec{E}=-\nabla V$$

where these equations should be utilized at the unknown potential nodes to compute the electrostatic potential V; after that, the electric field strength E can be easily calculated with Equation (9).

Table 6 presents the technical data for the modeled transformer [40]. Based on that, the building of an accurate model of the transformer is very difficult, so some simplifications were considered in this work: Only one winding was considered, and the tank in the model was considered to be a perfect cylinder equivalent to one-third of the real transformer tank [41]. Moreover, an approximately 10% voltage drop across transformer windings was considered due to the copper resistance of these windings that can slightly change the electric field stress along the winding [40]. Hence, from Section 2 and Section 3, all measured parameters of the NEO–MO mixture are used as input data for the simulation model to evaluate the distribution of the electrostatic field inside the transformer when using these oil mixtures.

**Figure 12:**
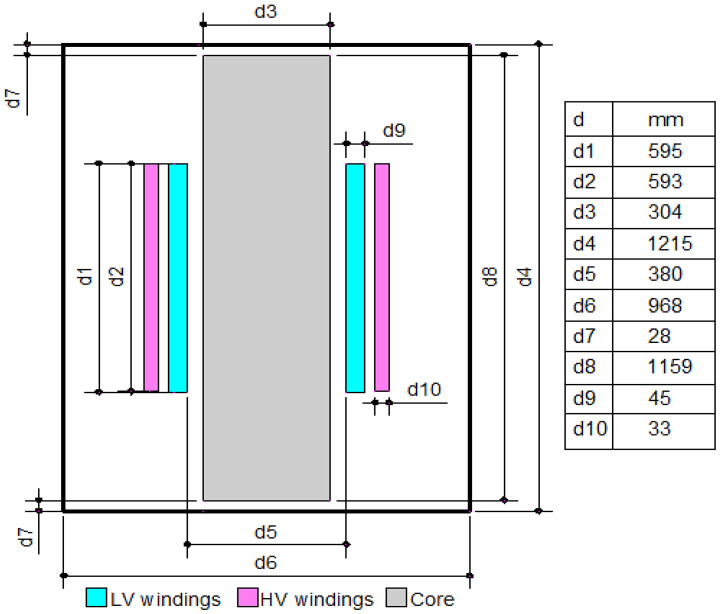
Two-dimensional geometry of the studied model (all dimensions in mm).

**Figure 13:**
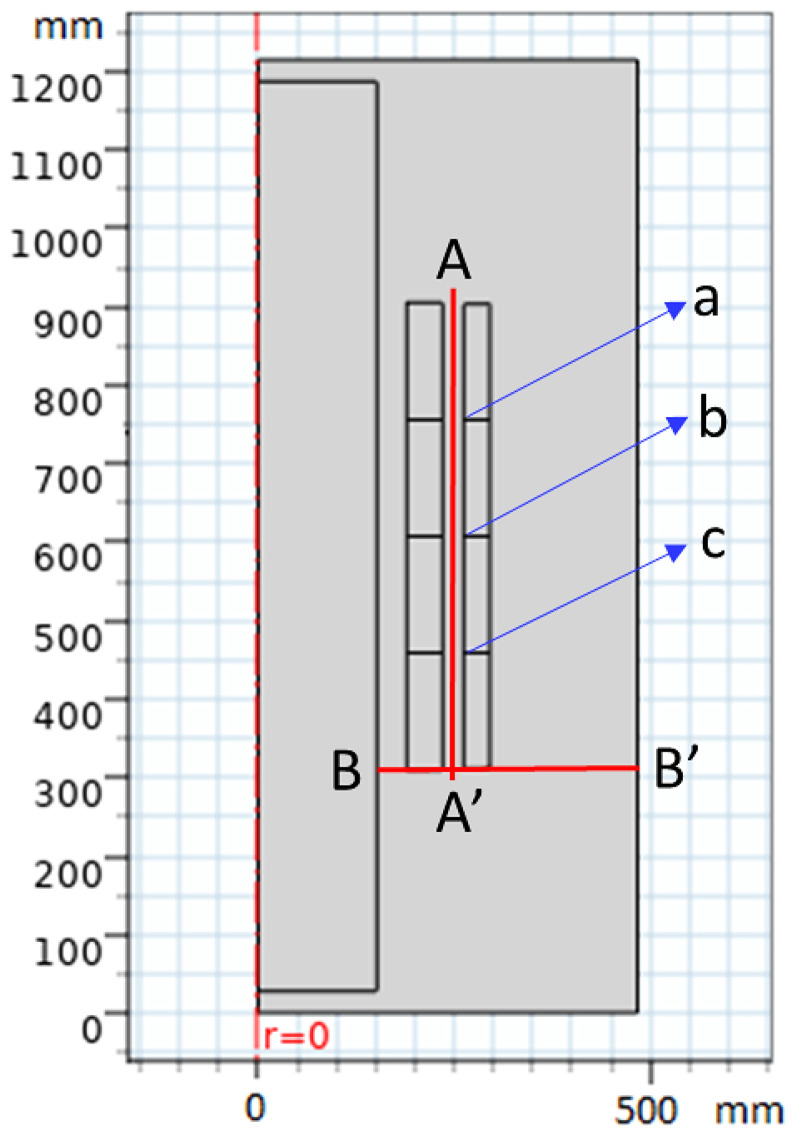
View of geometry creation of the model on COMSOL Multiphysics with millimeter dimensions for points (a, b, c) and line sections (AA’ and BB’).

**Figure 14:**
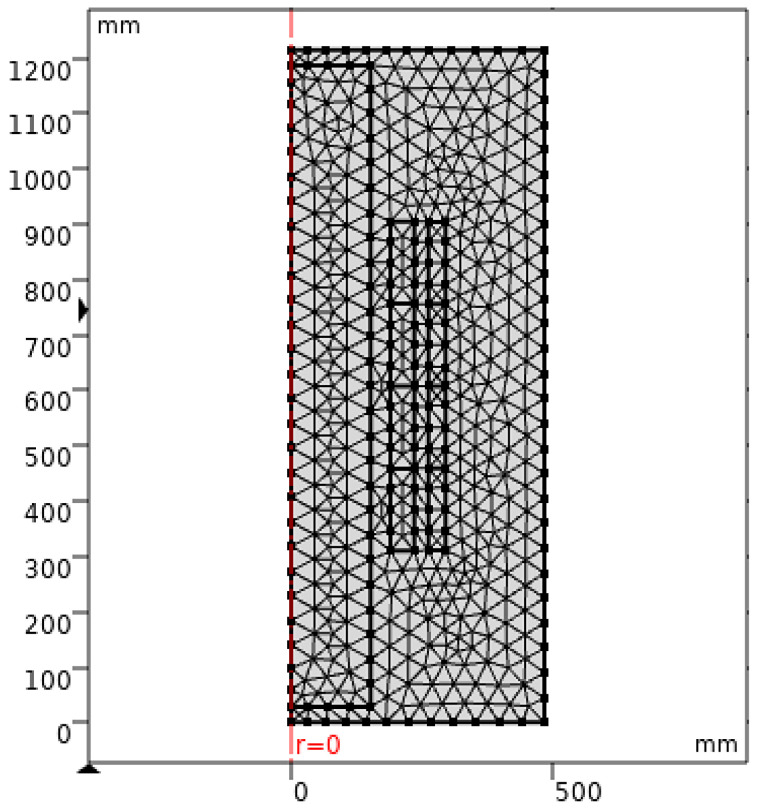
Model after the creation of the meshes for the finite element method solution (finer meshes style).

**Figure 15:**
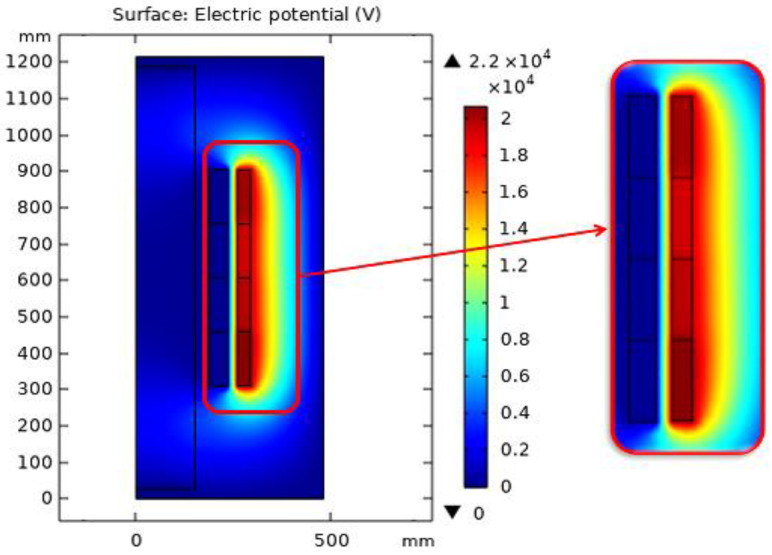
AC potential distribution inside all transformer parts based on FEM.

**Figure 16:**
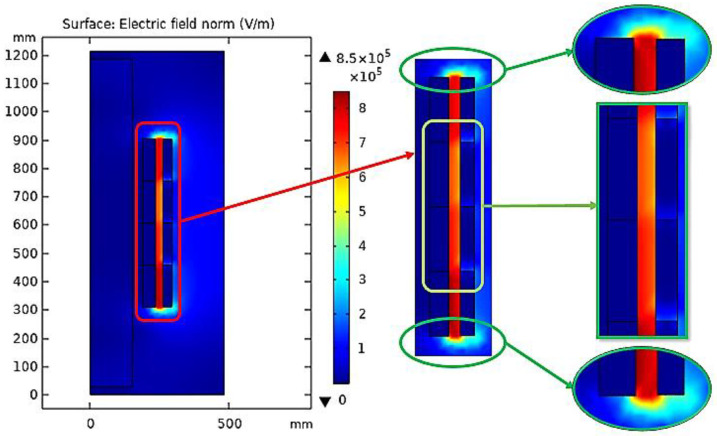
Electric field distribution inside the modeled transformer based on FEM.

When COMSOL Multiphysics is started, the model navigator enables the user to begin the modeling process and control all program settings. The space dimension is selected as 2D Axis-symmetric, and the application mode of electrostatic is adopted to begin working on the considered model. The model was created based on the dimensions shown in Figure 12. Moreover, Figure 13 presents the model after creation with the space dimension selected as 2D Axis-symmetric. Due to the build of the model under the electrostatic study [42], the AC voltage distribution inside all parts of the transformer can be obtained as shown in Figure 14. Furthermore, the electric field distribution can be evaluated based on the FEM using finer grid meshes as presented in Figure 15. Additionally, Figure 16 introduces the electric field distribution inside the modeled transformer, specifically at lines AA’ and BB’ and at points a, b, and c (see Figure 13). From the obtained results, it is clear that the electric field’s highest value points are concentrated between the low-voltage (LV) and high-voltage (HV) windings. These stresses are due to the higher voltage of HV winding compared to LV winding.

**Figure 17:**
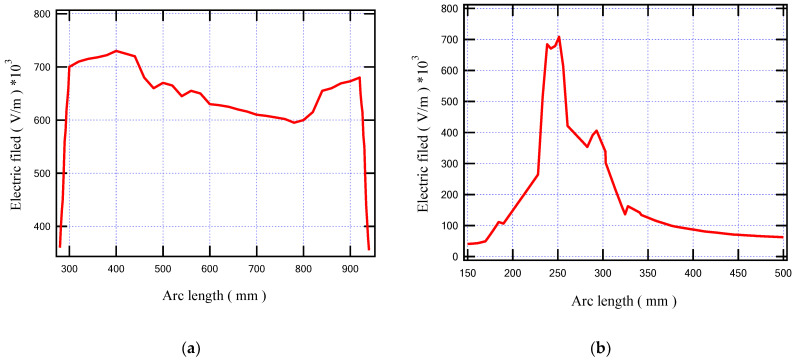
Electric field inside the modeled transformer along (**a**) line AA’ and (**b**) line BB’.

**Figure 18:**
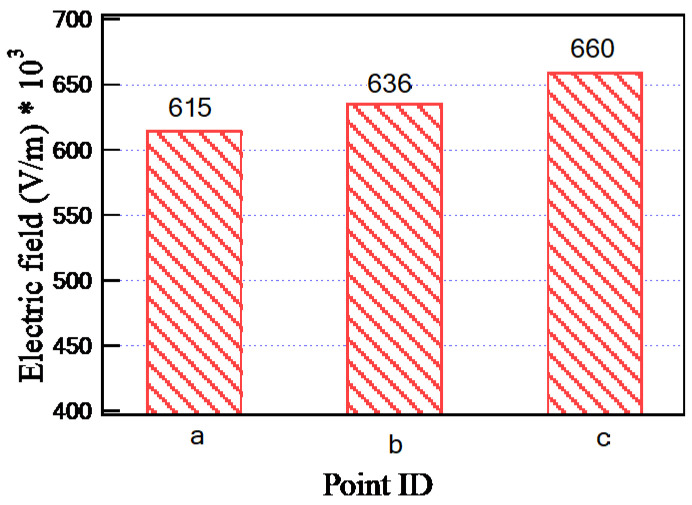
Electric field inside the modeled transformer at points a, b, and c.

To illustrate the values of the electric field in many locations on the insulating oil, the distribution of the electric field is evaluated along the lines AA’ and BB’ presented in Figure 13. The obtained electric field values along these lines are presented in Figure 17. Based on the obtained results, along AA’ that lies midway between the HV and LV windings, the higher electric field is presented nearly to the edges of the transformer windings at vertical arc lengths of 310 mm and 905 mm with electric fields of 705 kV/m and 678 kV/m, respectively. On the other hand, along BB’, the higher electric field is presented at a horizontal arc length of 251 mm with an electric field of 706 kV/m. Hence, the highest value points of the electric field on the insulating oil are presented at the edges of the transformer windings due to the direct interface between the transformer winding and the insulating oil molecules in this region. Accordingly, the best insulating oil is (10% MO6D + 90% NEO) from the transformer retrofilling point of view, which produces a good insulation and cooling performance via these highest value points compared to the other oil samples. Moreover, the transformer will have a longer lifetime, better performance, and fewer technical problems. Moreover, Figure 18 introduces the electric field at points a, b, and c (see Figure 13) that present a slight variation in the electric field due to the voltage drop along the transformer windings. The obtained results concluded that the electric field was varied at the selected points a, b, and c with values of 615, 636, and 660 kV/m, respectively, which do not exceed 10% variations in the electric field stress along the transformer winding.

## 6. Conclusions

This paper studied the dielectric properties and coolant behavior based on the dynamic viscosity of the transformer oil due to the replacement of worn-out mineral oil with natural ester oil. The experimental setup, measurements, and electrostatic simulation were introduced. From the above-mentioned results, the following remarks can be concluded:

- The mixing of NEO with aged MO leads to an enhancement in some characteristics of the mixed insulating oil, such as the dielectric strength and relative permittivity.
- The aged mineral oil has degraded dielectric and coolant properties when compared with the fresh mineral oil because of the electrical as well as thermal stresses on the oil molecules and bonds.
- The larger the amount of NEO in the mixed insulating oil, the higher the BDV due to the higher dielectric strength of the NEO that was mixed with the aged MO.
- The values of the dissipation factor for the mixed insulating oils exhibited a slight increase, but their values were kept under acceptable limits.
- The relative permittivity values for the mixed insulating oils were increased due to the increase in the percentage of NEO, which was due to the high dipole polarization inside the mixed oil.
- The dynamic viscosity of MO is lower than that of NEO for all ranges of temperature. This indicates that the cooling performance of MO is better than that of NEO.
- The electrostatic simulation of the oil transformer introduced the electric field on the insulating oil with higher values at the edges of the transformer windings due to the direct interface between the transformer winding and the insulating oil molecules in this region. Also, the variation in electric field stress on the transformer winding was taken into consideration for a more accurate model that did not exceed 10%.
- The best insulating oil (10% MO6D + 90% NEO) produces an optimal parameter for insulation and cooling performance compared to the other oil samples. Moreover, the transformer would have a longer lifetime, better diagnostic performance, and fewer technical problems.

In future work, the oil mixture performance under impulse waveforms or other fast transients will be investigated; in addition, the breakdown analysis at the paper/pressboard oil interface will be studied.

## Figures and Tables

**Table 1:** Specifications of natural ester oil and mineral oil.

Characteristic	Specification	
Oil type	MO	NEO
Appearance	Clear, transparent	Clear, light green
Density at 20 °C	0.88 (kg/cm^3^)	0.92 (kg/cm^3^)
Flash point	140 (°C)	325 (°C)
Pour point	−57 (°C)	−21 (°C)
Electric strength	>60 (kV)	>60 (kV)
Viscosity at 40 °C	9.4 (mm^2^/s)	<40 (mm^2^/s)
Total acidity	<0.01 (mg KOH/g)	<0.06 (mg KOH/g)
Water content	<20 (ppm)	<200 (ppm)

**Table 2:** Definitions of the variables of the relative permittivity (ε_r_) and dissipation factor (tan δ) equations.

Variable	Definition
$\varepsilon^{\prime }$	Dielectric coefficient
$\varepsilon^{\prime \prime }$	Dielectric loss
d	Gap spacing between the test electrodes
$C_{p}$	Oil sample capacitance
$\varepsilon_{o}$	Permittivity of free space
A	Test electrode cross-sectional area
f	Frequency
$R_{p}$	Oil sample resistance

**Table 3:** Description of the oil-tested samples.

ID	Description
Case (1)	Pure mineral oil aged for 6 days (MO6D)
Case (2)	Pure mineral oil aged for 12 days (MO12D)
Case (3)	Mixture of MO aged for 6 days 10% + fresh NEO 90% (10% MO6D + 90% NEO)
Case (4)	Mixture of MO aged for 6 days 20% + fresh NEO 80% (20% MO6D + 80% NEO)
Case (5)	Mixture of MO aged for 12 days 10% + fresh NEO 90% (10% MO12D + 90% NEO)
Case (6)	Mixture of MO aged for 12 days 20% + fresh NEO 80% (20% MO12D + 80% NEO)

**Table 4:** Weibull distribution analysis results for all insulating oil samples.

Oil Sample	ξ	*λ* (kV)	BDV 50% (kV)	BDV 10% (kV)
Fresh MO	9.8	67	64.6	53.3
Fresh NEO	83.6	101	100.4	98.2
MO6D	9.3	32.4	31.2	25.5
MO12D	10.8	30.2	29.2	24.6
**10% MO6D + 90% NEO**	**9.8**	**95.5**	**92**	**76**
20% MO6D + 80% NEO	5.7	86.5	81.1	58.2
10% MO12D + 90% NEO	6.7	95.7	90.5	68.3
20% MO12D + 80% NEO	7.7	68.5	65.3	51.2

**Table 5:** Dielectric properties for all insulating oil samples at a frequency of 50 Hz.

Oil Sample	*ε_r_*	tan *δ* (%)
Fresh MO	1.914	0.146
Fresh NEO	2.685	0.220
MO6D	1.925	0.149
MO12D	2.026	0.166
10% MO6D + 90% NEO	2.829	0.179
20% MO6D + 80% NEO	2.720	0.197
10% MO12D + 90% NEO	2.814	0.169
20% MO12D + 80% NEO	2.716	0.191

**Table 6:** Technical data for the modeled transformer [40].

Item	Specs
Type	Oil-filled
Standard specification	IEC 60076
Rated output at 45 °C ambient (kVA)	1000
Method of cooling	ONAN
Oil type	Mineral oil
System of connection	DYn-11
Turns ratio	22,000/400
Resistance/phase for primary RHV (Ω)	4.23
Resistance/phase for secondary RLV (Ω)	5.11 × 10^−4^
Permissible symmetrical S.C current at L.V side terminals for 2 s (kA)	62.55
Primary voltage at normal tapping (V)	22,000
Corresponding secondary voltage at no load (V)	400
Full load current at LV side I_LV_ (A)	1443.4
Full load current at HV side I_HV_ (A)	26.24
Efficiency at (100% −75%) rated output (%)	98.9–99.1
Iron losses (W)	1222
Copper losses (W)	9450
Temperature rise at rated output above 45 °C ambient temperature	
At oil top level (°C)	45
Winding temperature (°C)	55
Core temperature (°C)	55

## Data Availability

The data presented in this study are available on request from the corresponding author.
